# Alternative evolution of a spheroidal colony in volvocine algae: developmental analysis of embryogenesis in *Astrephomene* (Volvocales, Chlorophyta)

**DOI:** 10.1186/s12862-016-0794-x

**Published:** 2016-11-09

**Authors:** Shota Yamashita, Yoko Arakaki, Hiroko Kawai-Toyooka, Akira Noga, Masafumi Hirono, Hisayoshi Nozaki

**Affiliations:** 1 Department of Biological Sciences, Graduate School of Science, University of Tokyo, 7-3-1 Hongo, Bunkyo-ku, Tokyo 113-0033 Japan; 2 Department of Frontier Bioscience, Faculty of Bioscience and Applied Chemistry, Hosei University, 3-7-2 Kajino-cho, Koganei-shi, Tokyo 184-8584 Japan

## Abstract

**Background:**

Volvocine algae, which range from the unicellular *Chlamydomonas* to the multicellular *Volvox* with a germ–soma division of labor, are a model for the evolution of multicellularity. Within this group, the spheroidal colony might have evolved in two independent lineages: Volvocaceae and the goniacean *Astrephomene. Astrephomene* produces spheroidal colonies with posterior somatic cells. The feature that distinguishes *Astrephomene* from the volvocacean algae is lack of inversion during embryogenesis; the volvocacean embryo undergoes inversion after successive divisions to orient flagella toward the outside. The mechanisms of inversion at the molecular and cellular levels in volvocacean algae have been assessed in detail, particularly in *Volvox carteri*. However, embryogenesis in *Astrephomene* has not been subjected to such investigations.

**Results:**

This study relied on light microscopy time-lapse imaging using an actively growing culture of a newly established strain to conduct a developmental analysis of *Astrephomene* as well as to perform a comparison with the similar spheroidal volvocacean *Eudorina*. During the successive divisions involved in *Astrephomene* embryogenesis, gradual rotation of daughter protoplasts resulted in movement of their apical portions toward the embryonic posterior, forming a convex-to-spheroidal cell sheet with the apical ends of protoplasts on the outside. Differentiation of the posterior somatic cells from the embryo periphery was traced based on cell lineages during embryogenesis. In contrast, in *Eudorina*, the rotation of daughter protoplasts did not occur during successive cell divisions; however, inversion occurred after such divisions, and a spheroidal embryo was formed. Indirect immunofluorescence microscopy of basal bodies and nuclei verified this difference between *Astrephomene* and *Eudorina* in the movement of embryonic protoplasts.

**Conclusions:**

These results suggest different tactics for spheroidal colony formation between the two lineages: rotation of daughter protoplasts during successive cell divisions in *Astrephomene*, and inversion after cell divisions in *Eudorina*. This study will facilitate further research into the molecular and genetic mechanisms of the parallel evolution of the spheroidal colony in volvocine algae.

**Electronic supplementary material:**

The online version of this article (doi:10.1186/s12862-016-0794-x) contains supplementary material, which is available to authorized users.

## Background

The evolutionary origin of multicellularity is a topic of increasing interest. The evolution from unicellular to multicellular organisms is one of the evolutionary transitions in individuality (i.e., the integration of individuals into a new higher-level individual), which bring about diversification and the hierarchical organization of the biosphere [1]. The emergence of multicellularity occurred in at least 25 eukaryotic lineages independently [2]. However, almost all the multicellular lineages lack extant transitional states from unicellular ancestors, which hampers determination of the initial stages of multicellularity.

**Fig. 1:**
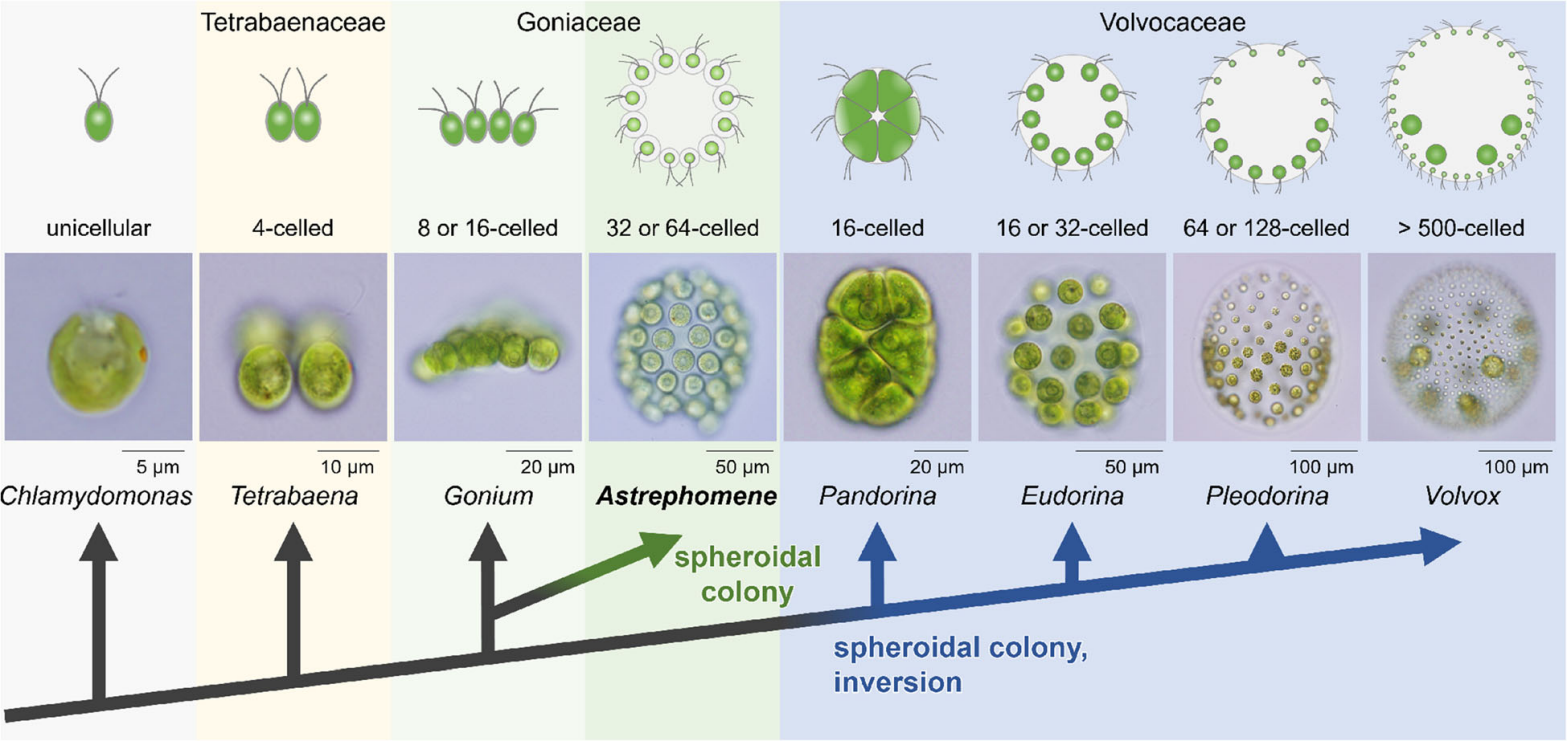
Schematic representation of the phylogenetic relationships of the volvocine algae and the parallel evolution of the spheroidal colony. Volvocine algae range from the unicellular *Chlamydomonas* to the multicellular *Volvox* through various intermediate forms and are used as a model for research into the evolution of multicellularity [3]. The spheroidal colony is thought to have evolved twice independently within this group [4, 5, 11]: once in the Volvocaceae, from *Pandorina* to *Volvox*, and the other in the genus *Astrephomene*. The phylogeny is based on previous reports [4–6]. All drawings and photographs represent side views of individuals with anterior ends orienting toward the top of this figure. All photographs are original

Volvocine algae (Fig. 1), *Volvox* and its relatives, are a model lineage for research into the evolutionary pathway from unicellular to multicellular organisms [3]. This lineage consists of various intermediate forms from unicellular *Chlamydomonas reinhardtii* to multicellular *Volvox* with a germ–soma division of labor. The phylogenetic relationships within this group have been resolved [4], and the gain or loss of characteristics related to multicellularity in ancestors has been deduced [5, 6]. Additionally, as *Chlamydomonas reinhardtii* and *Volvox carteri* are used as model organisms, their genome sequence data [7, 8] and diverse tools—such as for cultivation, molecular biology, and genetics—are available. These techniques are also applicable to other volvocine genera, and the initial steps in the evolution of multicellularity are being elucidated at the morphological [9], genetic, and genomic [10] levels. Further comparative analyses of volvocine algae should reveal further details of the evolution of multicellularity at the molecular and genetic levels.

Recent phylogenetic studies of the volvocine lineage have suggested that the spheroidal colony might have evolved from a flattened ancestor in two independent lineages: Volvocaceae and the goniacean *Astrephomene* [4, 5, 11]. Although the spheroidal colonies of these two lineages resemble each other in terms of their external morphology, there is a crucial difference between them in modes of colonial development or embryogenesis. Volvocaceae species undergo the drastic morphogenetic process known as inversion, whereas *Astrephomene* species do not [4, 5, 11].

Inversion is common among volvocacean species [4, 5, 11]. Each reproductive cell or gonidium undergoes successive cell divisions to form a concave-to-cup-shaped embryo or plakea composed of a single cell layer. Immediately after the successive divisions (“palintomy” or rapid multiple fissions without cell growth [12]), the cell layer is inside out compared with the adult configuration—the apical ends of the embryo protoplasts, in which basal bodies are localized and flagella are formed, are oriented toward the interior of the plakea. Therefore, the embryo undergoes inversion, during which the cell layer is inverted to form a spheroidal daughter colony with the apical ends and flagella of daughter protoplasts positioned outside. This process enables appropriate locomotion of spheroidal colonies of the Volvocaceae. The mechanism of inversion has been investigated extensively at the cellular and molecular levels using a model species, *Volvox carteri* [13–17]. Compared with that of *Volvox*, inversion in other volvocacean genera—such as *Pandorina*, *Eudorina*, and *Pleodorina*—is relatively simple [18–23]. Inversion in these volvocacean species has been examined by light microscopy time-lapse imaging [23] as well as by electron microscopy [18–20].

However, the developmental tactic of the volvocacean species is not applicable to other lineages of volvocine algae that form spheroidal colonies (Fig. 1). *Astrephomene* has 32- or 64-celled spheroidal colonies resembling those of some volvocacean species, but there is a difference in the distribution of somatic cells; *Astrephomene* has two or four somatic cells distributed only in the posterior pole of the colony [24, 25] whereas volvocacean species lack somatic cells or have somatic cells distributed in the anterior pole of the colony. The feature of *Astrephomene* that distinguishes them from the Volvocaceae is the lack of inversion during embryogenesis; each reproductive cell in a colony divides successively to form a spheroidal daughter colony [24, 26]. The embryogenesis of *Astrephomene* has been visualized by light microscopy in previous studies [24–26]. However, successive observations by light microscopy time-lapse imaging and cell-based studies of spheroidal colony formation in *Astrephomene* have not been performed; thus, the mechanism underlying the formation of a spheroidal colony without inversion is unclear.

The present study was undertaken to evaluate the cellular or subcellular mechanisms underlying the formation of spheroidal colonies of *Astrephomene*; to this end, we used light microscopy time-lapse imaging of an actively growing culture of a newly established strain and compared it with that of a volvocacean *Eudorina*, which has a similar cell number and colony size. The developmental events observed by time-lapse imaging were verified at the subcellular level by tracing the behavior of the basal bodies and nuclei of daughter protoplasts by indirect immunofluorescence microscopy. These results will facilitate further research into the cellular and molecular bases of the parallel evolution of spheroidal colony formation within the volvocine algae.

## Results

### Time-lapse imaging of embryogenesis in *Astrephomene*

**Fig. 2:**
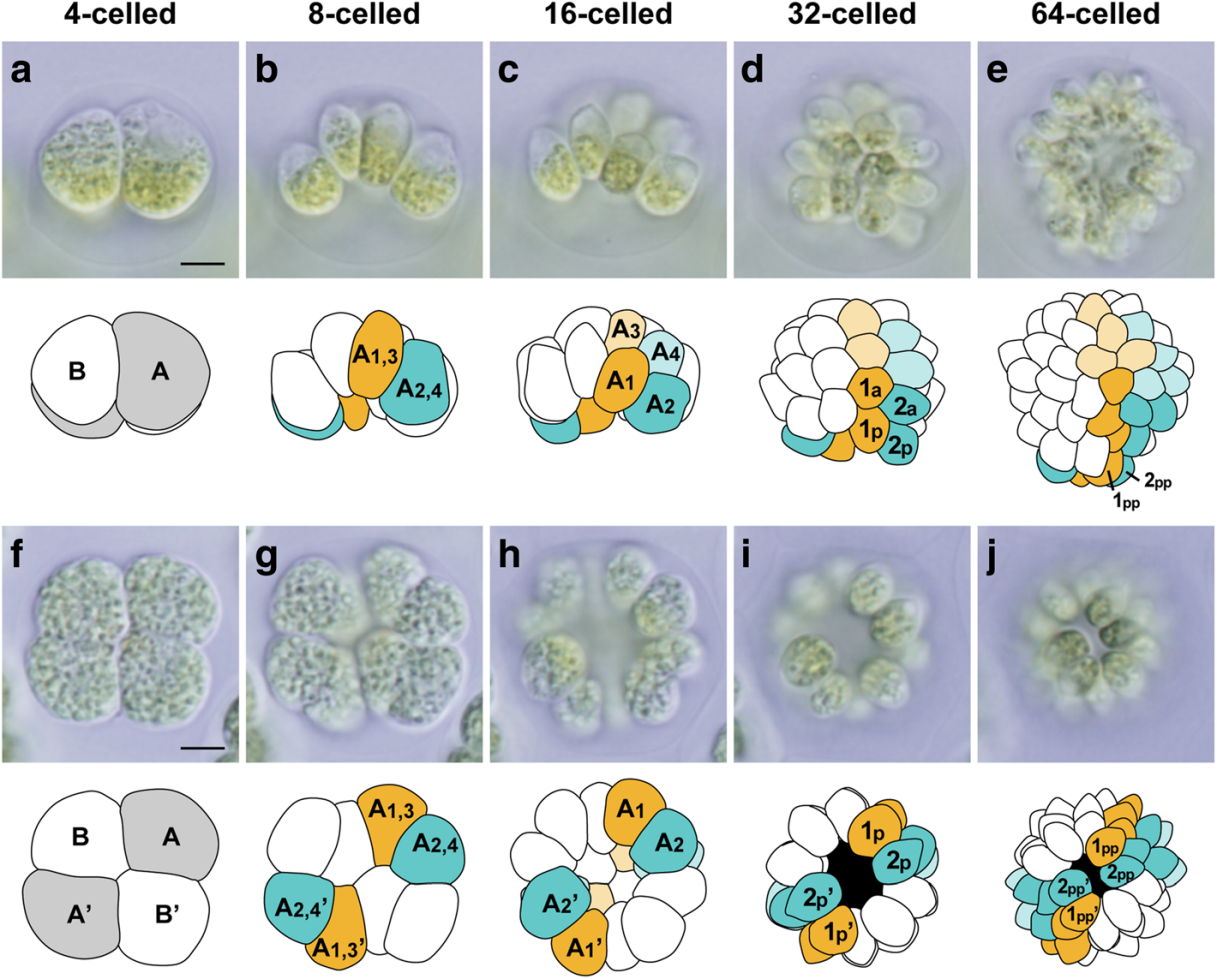
Cleavage patterns and cell lineages involved in embryogenesis of *Astrephomene*. Based on time-lapse analyses of two views of embryos (Additional files 2 and 4). Images in each row are shown at the same magnification. Scale bars: 5 μm. Successive lateral view **a**–**e** and posterior view **f**–**j** images are shown, together with diagrams. Outlines of daughter protoplasts were traced from images in different focal planes taken during a 1-min period. The cleavage pattern was 180° rotationally symmetrical about the longitudinal axis of the embryo. Four posterior somatic cells in the newly formed colony (1 pp, 2 pp, 1 pp’, and 2 pp’ cells in **e**, **j**) were derived from two diagonally opposite cells in four-celled embryos (A and A’ cells in **a**, **f**) and four peripheral cells in 16-celled embryos (A1, A2, A1’, and A2’ cells in **c**, **h**)

**Fig. 3:**
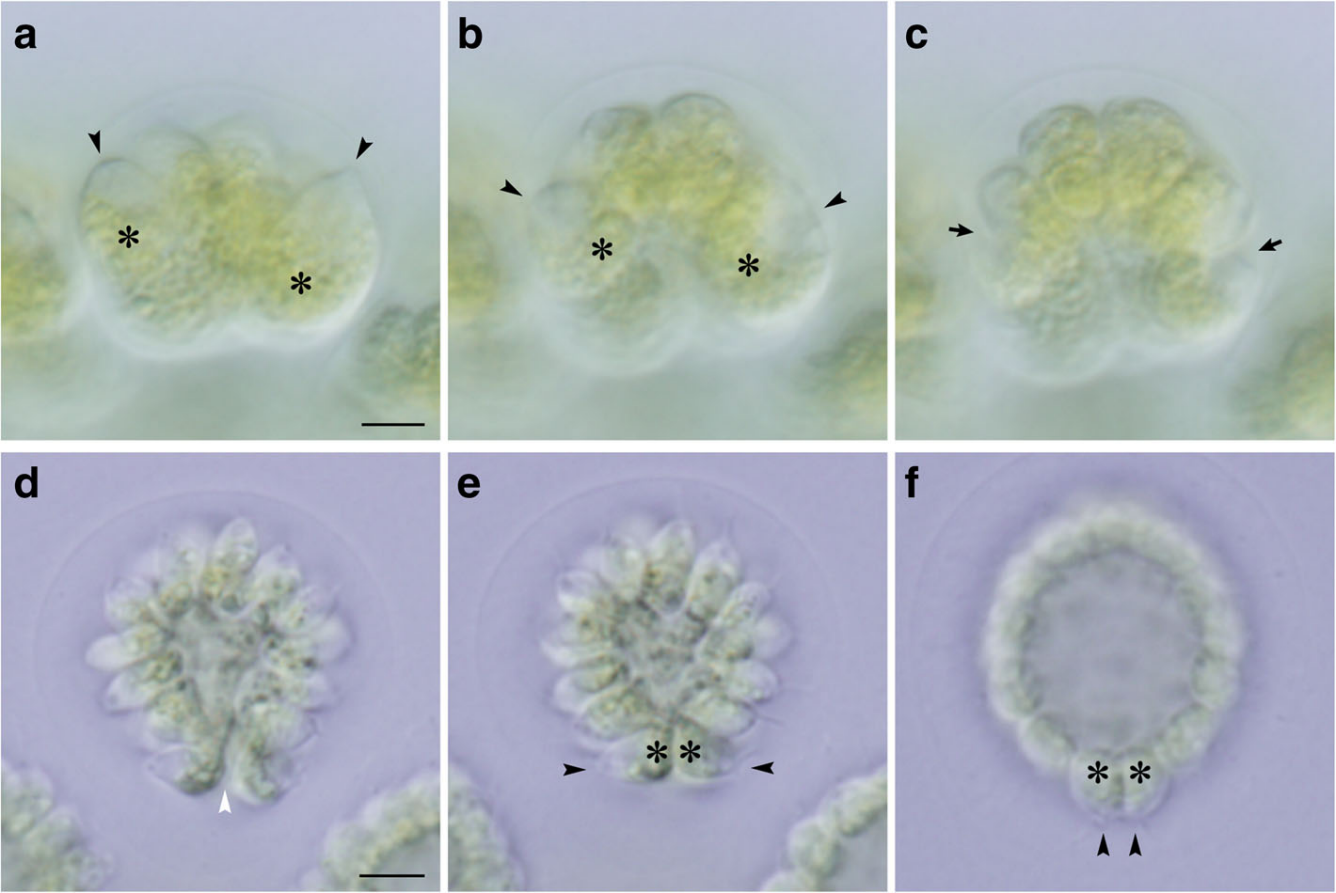
Behavior of daughter protoplasts during embryogenesis in *Astrephomene*. Time-lapse analyses of lateral views of embryos (Additional files 1 and 3). Images in the same row were obtained using the same magnification. Scale bars: 5 μm. **a**–**c** Successive anterior–lateral view images of an embryo at the 8-celled stage (**a**), late 8-celled stage (**b**), and early 16-celled stage (**c**). Note that the rotation of daughter protoplasts is indicated by the positions of the apical ends (*arrowheads*) and chloroplasts (*asterisks*) (**a**, **b**). The positions of cleavage furrows (*arrows*) corresponded approximately to the positions of the apical ends in the preceding stage (**b**, **c**). **d**–**f** Successive posterior–lateral view images of an early 64-celled embryo immediately after the final cleavage (**d**) and 5 min after the final cleavage (**e**), and an expanded 64-celled embryo or daughter colony (**f**). The posterior gap of the cell layer (*white arrowhead*, **d**) is closed soon after the last cleavage, and the shape of the daughter protoplasts changes slightly. The orientation of the anterior ends of posterior somatic cells (*arrowheads*, **e**, **f**) changes from lateral to posterior


Embryogenesis of a newly established *Astrephomene* strain, *Astrephomene gubernaculifera* 2014-1002-YkAs8, was observed by light microscopy time-lapse imaging. Time-lapse images of the anterior–lateral view (Additional file 1), lateral view (Additional file 2), posterior–lateral view (Additional file 3), and posterior view (Additional file 4) were obtained and analyzed as successive images and movies. During embryogenesis, each reproductive cell performed multiple divisions to form a spheroidal colony, as reported previously [24–26]. Moreover, cell divisions of all daughter protoplasts were synchronized, and six synchronous cell divisions resulted in the formation of 64-celled daughter colonies (Fig. 2). The intervals between cell divisions were approximately 20–30 min. Cleavage patterns were essentially identical in all reproductive cells. The cleavage patterns and cell lineages of embryogenesis in *Astrephomene* were traced by comparing images of the embryos obtained using different optical sections and angles (see Light microscopy time-lapse imaging in Methods) (Figs. 2 and 3).

Additional file 1: Movie S1. Time-lapse analysis of anterior-lateral view of embryogenesis in *Astrephomene*. Note the rotation of daughter protoplasts during successive cell divisions. Scale bar: 5 μm, 900x speed. (AVI 2600 kb)
Additional file 2: Movie S2. Time-lapse analysis of lateral view of embryogenesis in *Astrephomene*. Scale bar: 5 μm, 900x speed. (AVI 2160 kb)
Additional file 3: Movie S3. Time-lapse analysis of posterior-lateral view of embryogenesis in *Astrephomene*. Note the change of direction in four posterior somatic cells after successive cell divisions. Scale bar: 5 μm, 900x speed. (AVI 2420 kb)
Additional file 4: Movie S4. Time-lapse analysis of posterior view of embryogenesis in *Astrephomene*. Note the posterior gap surrounding by 4 cells (Fig. 2j; 1 pp, 2 pp, 1 pp’ and 2 pp’ cells) closed just after the sixth cell divisions. Scale bar: 5 μm, 900x speed. (AVI 2510 kb)


Based on the present time-lapse imaging, gradual rotation of daughter protoplasts was observed during successive divisions. The first two cleavages did not involve rotation of protoplasts and were similar to those in other volvocine algae [23]; this resulted in the generation of a four-celled embryo (Fig. 2a, f), which is typical of the colonies of volvocine algae [11], with two diagonal protoplasts attached to each other in the center (Fig. 2f). The rotation of daughter protoplasts was observed after the second division and occurred in conjunction with movement of the outer surface of the cell layer or the apical ends of daughter protoplasts toward the posterior of the embryo. In an optical section from the lateral side of the embryo, daughter protoplasts were positioned at the right side of the embryo rotated clockwise, whereas protoplasts at the left side were rotated counterclockwise (Fig. 3a, b). Immediately prior to the third cleavage, each daughter protoplast underwent slight rotation, which resulted in the formation of a concave eight-celled embryo with outside apical ends after the third cleavage (Fig. 2b, g). The rotation of daughter protoplasts was most marked immediately before the fourth cleavage (Fig. 3a–c); the apical ends of protoplasts, which frequently exhibited acute apices opposite pale-green chloroplasts, moved from the anterior to the posterior direction of the embryo (Fig. 3a, b). Moreover, the angles between the fourth cleavage planes and the longitudinal axis of the embryo were larger than those for the planes of the third cleavage. A hemispherical 16-celled embryo was formed after the fourth cleavage (Figs. 2c, h and 3c). Protoplasts underwent lesser rotations prior to the fifth cleavage than before the fourth cleavage, which resulted in an almost spheroidal 32-celled embryo with a small gap at the posterior pole (Fig. 2d, i). A very slight rotation of the protoplasts occurred before the sixth cleavage, resulting in the formation of a 64-celled embryo with a small gap at the posterior pole (Fig. 2e, j).

Immediately after the sixth cleavage, daughter protoplasts narrowed and commenced to produce flagella, and the posterior gap closed (Figs. 2j and 3d, e). Daughter protoplasts subsequently became flattened, and the hollow spheroidal daughter colony expanded (Fig. 3d, e). Simultaneously, the orientation of the longitudinal axes of the four cells at the posterior pole of the embryo changed from lateral to posterior, and their apical ends became protruded (Fig. 3f). These four cells were elongate-ovoid or ellipsoidal in shape and slightly larger than the other daughter cells. The most posterior position and orientation of the longitudinal axes of the four cells corresponded to those in posterior somatic cells of mature vegetative colonies [24, 25] (see also Additional file 5: Figure S1); therefore, these four cells likely become somatic cells at maturity.

Based on images of the developing embryos obtained using different optical sections and angles, the cell lineage of somatic cells was traced. The pattern of cell divisions was 180° rotationally symmetrical about the longitudinal axis of the embryo, and the origins of four posterior somatic cells (Fig. 2e, j; 1 pp, 2 pp, 1 pp’, and 2 pp’ cells) were determined; the somatic cells were derived from two diagonally opposite daughter protoplasts in four-celled embryos (Fig. 2a, f; A and A’ cells) and through two opposite pairs of adjoining protoplasts at the periphery of 16-celled embryos (Fig. 2c, h; A1, A2, A1’ and A2’ cells).

**Fig. 4:**
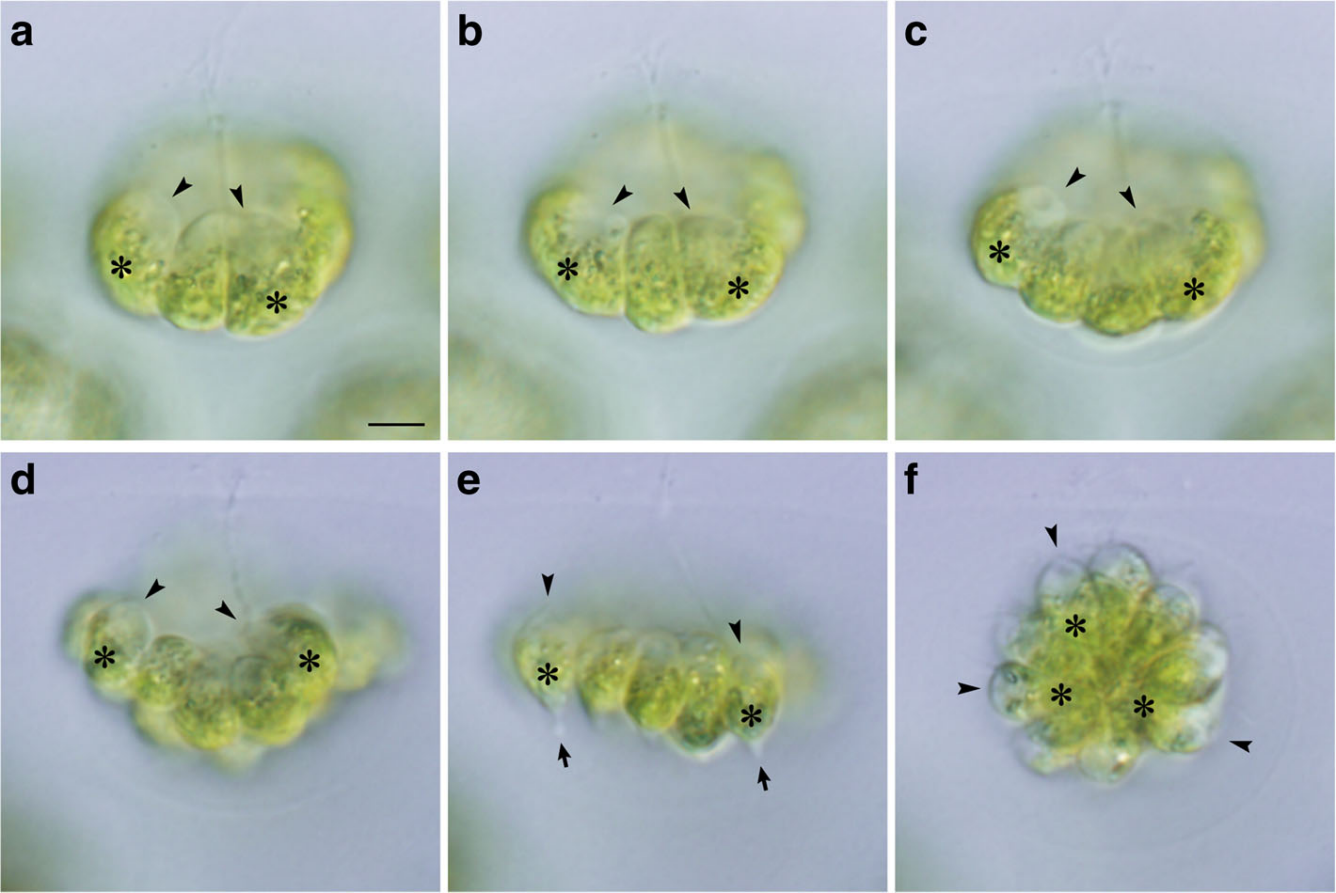
Cell divisions and inversion during embryogenesis in *Eudorina*. Successive stages of an embryo observed by time-lapse analysis from anterior-lateral view (Additional file 6). All at the same magnification throughout. Scale bar: 5 μm. Note the longitudinal axis of each daughter protoplasts indicated by positions of apical ends (*arrowheads*) and chloroplasts (*asterisks*). Rotation of daughter protoplasts is not observed during cell divisions (**a**–**c**). The concave surface of plakea or apical ends of the constitutive protoplasts (**d**) become outer surface of the spheroid by means of inversion (**e**, **f**). **a** Early 8-celled stage. **b** Late 8-celled stage. **c** Early 16-celled stage. **d** 32-celled stage before inversion. **e** Inverting plakea. Note formation of stalks (*arrows*) at the chloroplast ends of daughter protoplasts. **f** Spheroidal daughter colony just after inversion


To directly compare embryogenesis in the *Astrephomene* with that of the Volvocaceae, light microscopy time-lapse images of embryogenesis in *Eudorina* sp. were analyzed (Additional file 6). During formation of daughter colonies of *Eudorina*, most 32 or 16 vegetative cells divided five times successively to produce 32-celled concave plakeas, which became spheroidal daughter colonies by means of inversion, as reported previously [19, 20, 23]. The present time-lapse analyses of *Eudorina* demonstrated that rotation of daughter protoplasts did not occur during successive cell divisions (Fig. 4a, b), and the angles between the cleavage planes and the longitudinal axis of the mother protoplast did not change markedly (Fig. 4c). After five successive divisions, a concave plakea, a single cell layer within which were the apical ends of the protoplasts, was formed (Fig. 4d). After the final cleavage, inversion occurred to bend the cell layer to form a spheroidal daughter colony. Each daughter protoplast formed a stalk at the chloroplast end during the initial stage of inversion (Fig. 4e, f).

Additional file 6: Movie S5. Time-lapse analysis of anterior-lateral view of embryogenesis in *Eudorina*. Note absence of rotation of daughter protoplasts during successive cell divisions and inversion with stalk formation after cell divisions. Scale bar: 5 μm, 900x speed. (AVI 2440 kb)


### Indirect immunofluorescence microscopy of basal bodies and nuclei

The cellular behavior observed by light microscopy time-lapse imaging was verified at the subcellular level by indirect immunofluorescence microscopy of basal bodies and nuclei. For immunostaining of basal bodies, an antibody against *Chlamydomonas* SAS-6, which is localized in mature and immature basal bodies [27], was reacted with fixed embryos; thus, two basal bodies and two pro-basal bodies in each cell were observed as four dots. An anti-histone antibody was reacted with the samples at the same time, allowing simultaneous visualization of basal bodies and nuclei. The localization of basal bodies and nuclei was used to identify the longitudinal axis of daughter protoplasts during embryogenesis.

**Fig. 5:**
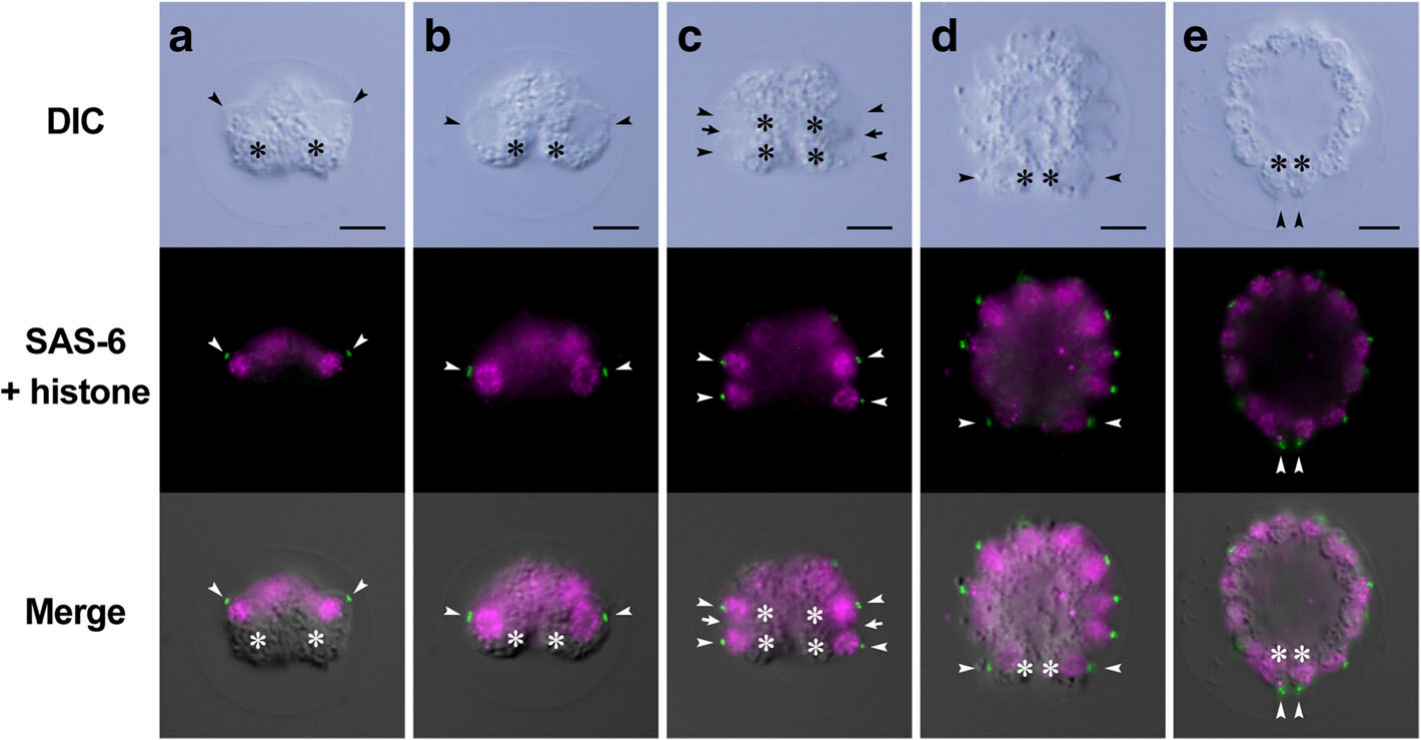
Indirect immunofluorescence microscopy of developing *Astrephomene* embryos. Differential interference contrast (DIC) images (*top row*), fluorescence images (*middle row*) labeled with anti-SAS-6 (*green*) and anti-histone (*magenta*) antibodies, and merged DIC and fluorescence images (*bottom row*) of the same embryos are shown. Scale bars: 5 μm. Positions of basal bodies labeled with an anti-SAS-6 antibody (*arrowheads*) and chloroplasts (*asterisks*) are shown. **a** Early 8-celled embryo. **b** Late 8-celled embryo. Note that the positions of the basal bodies of daughter protoplasts (*arrowheads*) are changed from the anterior to the posterior region of the embryo during the eight-celled stage. **c** Early 16-celled embryo. Note that the positions of the fourth cleavage furrows (*arrows*) correspond approximately to the positions of basal bodies at the 8-celled stage (**b**). **d** Early 64-celled embryo. **e** A 64-celled daughter colony. Note that the posterior somatic cells are oriented toward the posterior direction of the daughter colony (**d**)

The position and movement of basal bodies and nuclei in daughter protoplasts during the embryogenesis of *Astrephomene* confirmed the rotation of daughter protoplasts during successive divisions (Fig. 5). The developmental stages of fixed embryos were identified using the time-lapse images. As the cell divisions progressed, the basal bodies of the daughter protoplasts at the outer surface of the convex or hollow embryo moved from the anterior pole to the posterior pole of the embryo (Fig. 5a, b). The position of the basal bodies immediately after the rotation of protoplasts corresponded to the subsequent cleavage furrows (Fig. 5b, c). Additionally, the drastic change in the longitudinal axes of the four posterior somatic cells in the 64-celled stages observed by the time-lapse imaging (Fig. 3e, f) was verified by the positions of the basal bodies of the four most posterior protoplasts of the embryos (Fig. 5d, e).

**Fig. 6:**
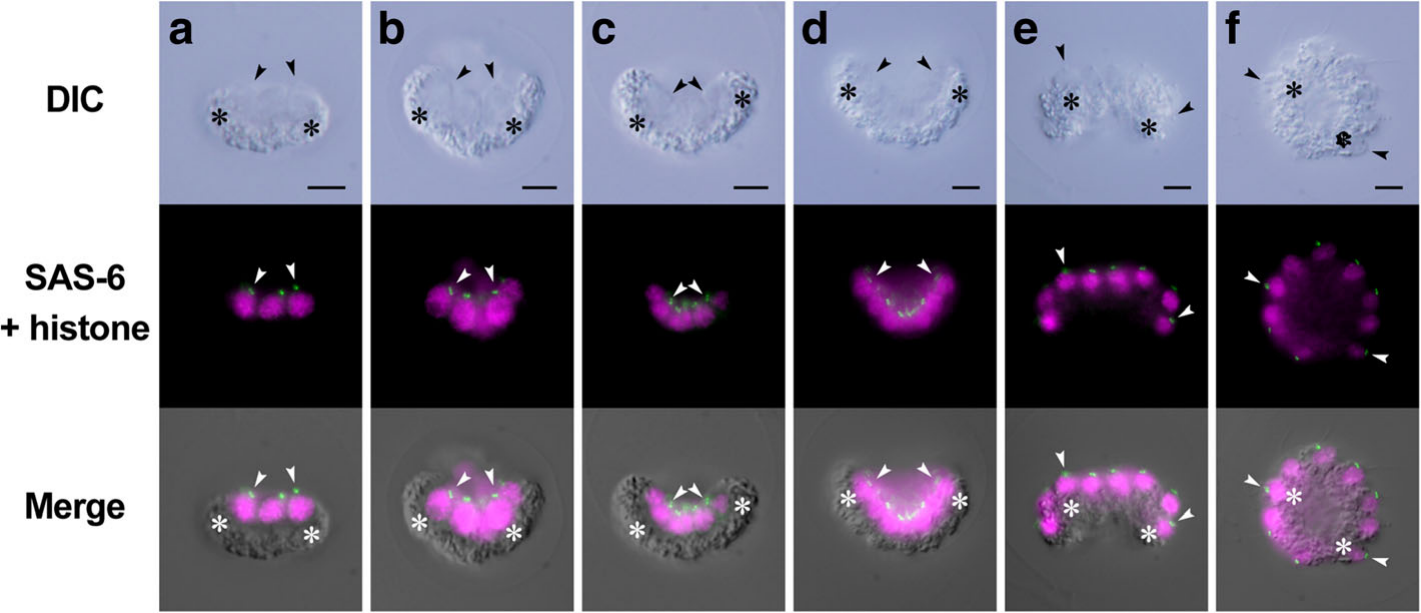
Indirect immunofluorescence microscopy of daughter colony formation in *Eudorina*. Differential interference contrast (DIC) images (*top row*), fluorescence images (*middle row*) labeled with anti-SAS-6 (*green*) and anti-histone (*magenta*) antibodies, and merged images of DIC and fluorescence images (*bottom row*) of the same embryos are shown lengthwise. Scale bars: 5 μm. Positions of basal bodies labeled with anti-SAS-6 antibody (*arrowheads*) and chloroplasts (**c**) are shown. Note that basal bodies of daughter protoplasts are positioned in the center of concave surface of plakea before inversion (**a**–**d**). Basal bodies are moved from interior or concave surface of embryo to the outer surface of the spheroidal daughter colony by inversion (**d**–**f**). **a** Early 8-celled embryo. **b** Late 8-celled embryo. **c** Early 16-celled embryo. **d** Early inversion stage of 32-celled embryo. Note that the periphery of the plakea commences bending toward outside and enlarging the anterior opening of the plakea. **e** Inverting 32-celled embryo. **f** 32-celled embryo after inversion

In contrast to the gradual change of basal body positions between cell divisions in *Astrephomene*, basal bodies moved only during inversion in the embryogenesis of *Eudorina* (Fig. 6). The basal bodies of the daughter protoplasts did not move markedly within the *Eudorina* embryo and remained at the concave face of the plakea during successive cell divisions (Fig. 6a–c). This situation is consistent with the fact that there was no rotation of daughter protoplasts between cell divisions in *Eudorina* (Fig. 4). Basal bodies inside the concave cell layer after successive cell divisions (Fig. 6d) came to be oriented toward the outside of the spheroidal cell layer due to inversion (Fig. 6e, f).

## Discussion

Using light microscopy time-lapse imaging of the embryogenesis of *Astrephomene* with a high temporal resolution (1 min) and several views, we demonstrated the dynamic cellular events, rotation of daughter protoplasts between cell divisions, and drastic change in the orientation of the posterior somatic cells after successive cell divisions. Time-lapse observation was required as the intervals between cell divisions were > 20 min, and rotational movement was slow. These results were verified by the indirect immunofluorescence microscopy of basal bodies and nuclei.

**Fig. 7:**
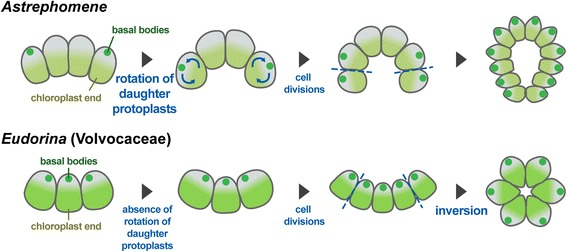
Schematic diagrams of the two mechanisms of spheroidal colony formation in the volvocine algae. In *Astrephomene*, rotation of daughter protoplasts occurs in conjunction with the movement of basal bodies during successive cell divisions. In *Eudorina*, protoplast rotation is lacking during successive divisions; a spheroidal colony is formed by means of inversion after successive divisions

Comparison of the cellular events during the embryogenesis of *Astrephomene* with those in that of volvocacean *Eudorina* revealed two distinct mechanisms of spheroidal colony formation (Fig. 7). For the appropriate locomotion of the spheroidal colony, the basal bodies of daughter protoplasts in the embryos of both lineages must be oriented toward the outside. In *Astrephomene*, the rotation of daughter protoplasts with the movement of basal bodies occurred between cell divisions, resulting in the formation of a convex-to-spheroidal cell layer with basal bodies outside (Figs. 3a–c and 5a–c). In contrast, we did not observe rotation of daughter protoplasts or movement of basal bodies during successive cell divisions in *Eudorina* (Figs. 4a–c and 6a–c). Inversion then occurred to form a spheroidal daughter colony with basal bodies outside (Figs. 4d–f and 6d–f), as in other members of the Volvocaceae [23]. Thus, the following mechanisms of spheroidal colony formation seem to have evolved in the two lineages: gradual rotation of protoplasts during successive divisions in *Astrephomene*, and inversion after successive divisions in the Volvocaceae.

Although the present study revealed the cellular mechanism of spheroidal colony formation in *Astrephomene* (i.e., rotation of daughter protoplasts during successive cell divisions), the underlying molecular mechanism is unclear. One candidate mechanism is an *InvA* homolog. During the inversion of *Volvox carteri*, InvA, a kinesin, is localized in the cytoplasmic bridges connecting daughter protoplasts and interacts with cortical microtubules to move cell bodies against the cytoplasmic bridges, which produces the force that drives the bending of the cell sheet [17]. As embryos of *Astrephomene* also have cytoplasmic bridges between daughter protoplasts [28], it is possible that an InvA homolog or a similar motor protein localized in cytoplasmic bridges interacts with cortical microtubules; this would occur between, rather than after, cell divisions to rotate daughter protoplasts. Further developmental analyses of *Astrephomene* should aim to identify the molecular mechanism underlying the rotation of daughter protoplasts in *Astrephomene*.

This study also demonstrated the cell lineages of posterior somatic cells (Fig. 2) and their change of direction toward the posterior pole after successive cell divisions (Fig. 3d–f). The volvocacean species *Pleodorina californica* has somatic cells at the anterior region in a spheroidal colony. These anterior somatic cells are derived from the daughter protoplasts in the center of a plakea during embryogenesis [21]. In *Astrephomene*, the posterior somatic cells were derived from the periphery of the embryo. These phenomena show that the correspondence of the central–peripheral polarity in embryos to the anterior–posterior axis in mature spheroidal colonies is conserved between the two lineages.

## Conclusion

Using light microscopy time-lapse imaging and indirect immunofluorescence microscopy of basal bodies and nuclei, we identified a difference in the cellular mechanism of spheroidal colony formation between *Astrephomene* and the Volvocaceae in the present study. The major cellular mechanisms of spheroid colony formation in *Astrephomene* and the Volvocaceae are rotation of the protoplast during successive cell divisions and inversion, respectively. However, these two mechanisms might be related to the homologous molecular mechanisms discussed above. Further research will reveal the molecular and genetic bases of the parallel evolution of the spheroidal colony in volvocine algae.

## Methods

### Strains and culture conditions

The *Astrephomene* strains established in previous research [25] showed abnormal morphology (not shown), possibly due to their long-term maintenance. Thus, a new strain of *Astrephomene* suitable for developmental observation was established in the present study. *Astrephomene gubernaculifera* strain 2014-1002-YkAs8 was isolated from a soil sample collected from a rice paddy field (35° 23’23.1” N, 140°01’36.2” E) in Yokota, Sodegaura, Chiba Prefecture, Japan on July 21, 2014. The dried soil was rewetted with boiled pea (*Pisum sativum*) and its extract in Petri dishes (90 × 20 mm) and incubated at 25 °C on a 12-h light/12-h dark schedule under cool-white fluorescent lamps at an intensity of 50–90 μmol · m^−2^ · s^−1^. After 3–4 days, vegetative colonies appeared in the Petri dishes. Clones were established using the pipette-washing method [29] and grown in 10 mL of VTAC medium [30] in screw-capped tubes (18 × 150 mm). The culture was maintained at 25 °C on a 12-h light/12-h dark schedule as described above. Species identification was carried out based on morphological and molecular data (Additional file 5: Figures S1 and S2).

For comparison with volvocacean species with similar colonial organization, *Eudorina* sp. strain 2010-623-F1-E8 was used. This is the F1 strain of *Eudorina* sp. NIES-2734 and NIES-2735 [31] and was used in a previous study [32].

For light microscopy time-lapse imaging, both species were grown in screw-capped tubes (18 × 150 mm) containing 10 mL VTAC medium under the culture conditions described above. For indirect immunofluorescent microscopy, to improve the synchrony, cultures were grown in silicon-capped 500 mL Erlenmeyer flasks containing 250 mL VTAC medium with aeration at 32 °C on a 16-h light/8-h dark schedule under cool-white fluorescent lamps at an intensity of 140–180 μmol · m^−2^ · s^−1^. Under these conditions, the asexual life cycle of *Astrephomene* was completed in approximately 24 h, and the culture was highly synchronized with the light–dark cycle. Almost all (99 %) colonies of *Astrephomene* initiated embryogenesis 6 h before the onset of the dark period. In *Eudorina*, the asexual life cycle was completed in approximately 48 h, and the culture was not highly synchronized with the light–dark cycle. Embryogenesis was initiated 1 or 2 h after the onset of the dark period in 30 % of the *Eudorina* colonies.

### Light microscopy time-lapse imaging

Embryogenesis of *Astrephomene gubernaculifera* 2014-1002-YkAs8 and *Eudorina* sp. 2010-623-F1-E8 was observed by time-lapse light microscopy based on a method reported previously [9] with some modifications (Additional file 5: Figure S3). To examine the embryos from anterior–lateral, lateral, posterior–lateral and posterior angles, fully mature vegetative colonies of *Astrephomene* were fragmented into several parts using a Dounce tissue grinder (Wheaton Industries Inc., Millville, NJ, USA) and attached to coverslips coated with polyethylenimine. Then, the coverslips were placed on slides and sealed with Vaseline. Preparations were observed using a BX-53 microscope (Olympus, Tokyo, Japan) equipped with Nomarski interference optics. Plural photomicrographs with different optical sections were obtained using DP Controller 1. 2. 1108 (Olympus) at 1-min intervals with manual successive changes in focus. In *Eudorina*, as fragmentation of the colonies was not possible, fully mature colonies were directly attached to coverslips and observed as described above. Only anterior–lateral view images were obtained from *Eudorina*. The images of both species were analyzed and processed using ImageJ 1.50b (National Institutes of Health, Bethesda, MD, USA).

### Indirect immunofluorescence microscopy

To verify the behavior of daughter protoplasts during the embryogenesis of *Astrephomene gubernaculifera* 2014-1002-YkAs8 and *Eudorina* sp. 2010-623-F1-E8, immunostaining of basal bodies and nuclei was performed as described previously [9], with the exception of the primary and secondary antibodies used to stain nuclei. A mouse monoclonal anti-histone H1 antibody (clone F152.C25.WJJ, Merck Millipore Corp., Darmstadt, Germany) diluted 1:500 with blocking buffer (0.44 % gelatin [Sigma-Aldrich, St. Louis, MO, USA], 0.05 % NaN_3_, 1 % BSA [Sigma-Aldrich], 0.1 % Tween 20 [Sigma-Aldrich] in phosphate-buffered saline) was used as the primary antibody to stain nuclei. The secondary antibody was an Alexa Fluor 568-conjugated goat anti-mouse IgG (Invitrogen, Carlsbad, CA, USA) diluted 1:500 with blocking buffer. Because a rabbit anti-CrSAS-6 antibody [27] and Alexa Fluor 488 goat anti-rabbit IgG (Invitrogen) were used as the primary and secondary antibodies, respectively, for staining basal bodies, the double staining of basal bodies and nuclei was feasible. Preparations were observed using a BX-60 microscope (Olympus) equipped with Nomarski interference optics, a mercury lamp, and filter sets with DP Controller 1.2.1108 (Olympus). Differential interference contrast images and fluorescence images were merged using Adobe Photoshop CC (Adobe Systems Inc., San Jose, CA, USA). To evaluate the specificity of the anti-CrSAS-6 antibody for *Astrephomene* and *Eudorina*, western blot analysis was carried out as described previously [27] (Additional file 5: Figure S4).

## Additional files

Additional file 5: Figure S1. Morphological observation of vegetative colonies in *Astrephomene gubernaculifera* strain 2014-1002-YkAs8. **Figure S2.** Maximum-likelihood tree of *Astrephomene* and *Gonium* based on *rbcL* genes. **Figure S3.** Diagram of making preparations for light microscopy time-lapse imaging of *Astrephomene* embryogenesis. **Figure S4.** Western blot of two species with anti-CrSAS-6 antibody. (PDF 688 kb)
