# Supplementary material for: Alternative evolution of a spheroidal colony in volvocine algae: developmental analysis of embryogenesis in Astrephomene (Volvocales, Chlorophyta)
Source: BMC Evol Biol. 2016 Nov 9;16:243. doi: 10.1186/s12862-016-0794-x (PMC5103382; doi:10.1186/s12862-016-0794-x)
Supplement: Additional file 5: Figure S1. — Morphological observation of vegetative colonies in Astrephomene gubernaculifera strain 2014-1002-YkAs8. Figure S2. Maximum-likelihood tree of Astrephomene and Gonium based on rbcL genes. Figure S3. Diagram of making preparations for light microscopy time-lapse imaging of Astrephomene embryogenesis. Figure S4. Western blot of two species with anti-CrSAS-6 antibody. (PDF 688 kb) [file 12862_2016_794_MOESM5_ESM.pdf]

Additional file 5:

Figure S1–S4

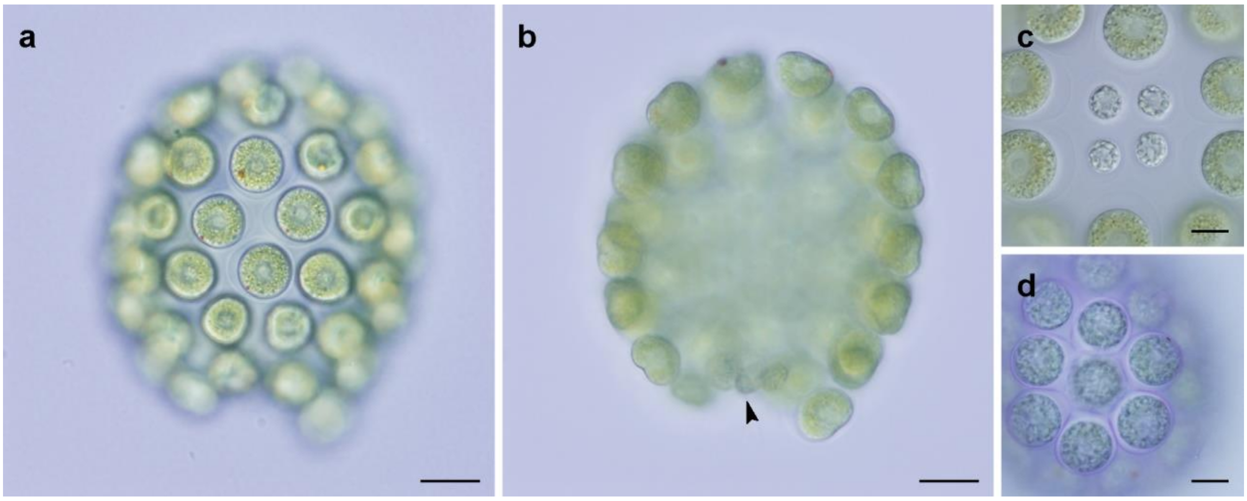

Figure S1 Morphological observation of vegetative colonies in *Astrephomene gubernaculifera* strain 2014-1002-YkAs8. Light microscopy of mature vegetative colonies in one-day-old culture was carried out with a BX53 microscope (Olympus, Tokyo, Japan) equipped with Nomarski interference optics. Based on the number of posterior somatic cells in 64-celled colonies and the characteristic of cellular sheaths [1], the *Astrephomene* strain 2014-1002-YkAs8 was identified as *Astrephomene gubernaculifera*. **a** Surface view of a 64-celled colony. Scale bar: 20 µm. **b** Median optical section of a 64-celled colony. Note small somatic cells at the posterior pole of colony (*arrowhead*). Scale bar: 20 µm. **c** Four somatic cells at the posterior pole of 64-celled embryo. Note the somatic cells are smaller and paler than reproductive cells. Scale bar: 10 µm. **d** Cellular sheaths stained with diluted methylene blue. Note cellular sheaths attached to each other without fenestrations. Scale bar: 10 µm.

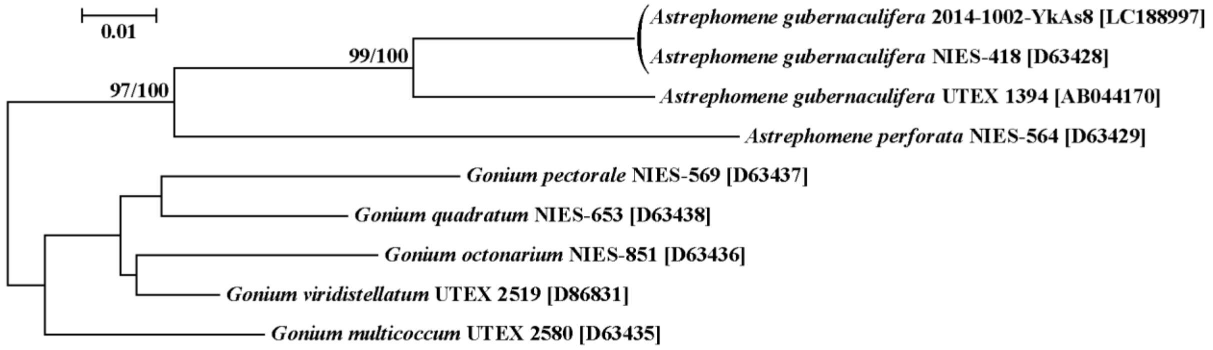

Figure S2 Maximum-likelihood tree of *Astrephomene* and *Gonium* based on *rbcL* genes. The coding region of *rbcL*, the chloroplast gene for the large subunit of RuBisCO, of *A. gubernaculifera* strain 2014-1002-YkAs8 were sequenced with essentially the same method in previous research [2–5]. The 1,128 bp corresponded to position 31-1,158 of *rbcL* from *Astrephomene* and *Gonium* (as outgroup) were subjected to maximum-likelihood analyses based on the GTR + G models with 1000 replicates of bootstrap analyses [6] performed by MEGA 6.06 [7]. As the sequence from *Astrephomene* strain 2014-1002-YkAs8 was completely the same as sequence from *A. gubernaculifera* NIES-418, they were treated as a single operational taxonomic unit. Accession numbers of sequence data were shown in brackets. Bootstrap value (≥ 50%) from maximum-likelihood (left), and neighbor-joining (right) are shown at branches.

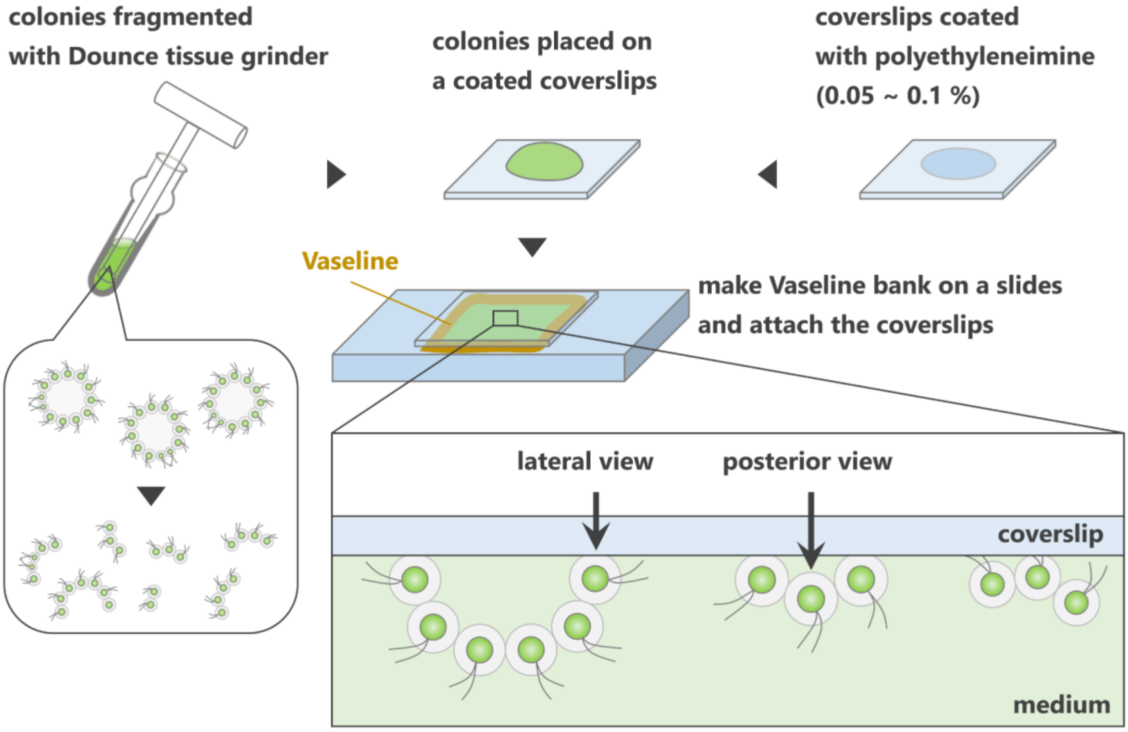

Figure S3 Diagram of making preparations for light microscopy time-lapse imaging of *Astrephomene* embryogenesis. In order to examine the embryos from anterior-lateral, lateral, posterior-lateral and posterior views, fully mature vegetative colonies of *Astrephomene* were fragmented into several parts with Dounce tissue grinder and attached to coverslips coated with polyethyleneimine. Then the coverslips were put on slides and sealed with Vaseline.

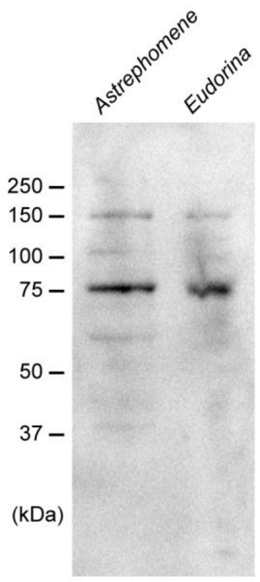

Figure S4 Western blot of two species with anti-CrSAS-6 antibody. The protein bands were detected with anti-CrSAS-6 antibody in *Astrephomene gubernaculifera* strain 2014-1002-YkAs8 and *Eudorina* sp. strain 2010-623-F1-E8. Western blotting analysis was carried out as described previously [8] for evaluation of the specificity of the antibody. Western blotting showed that the anti-CrSAS-6 antibody cross-reacted with SAS-6 from the two species.

References for Additional file 5

1. Nozaki H. Morphology and taxonomy of two species of *Astrephomene* (Chlorophyta, Volvocales) in Japan. J Jpn Bot. 1983;58:345–52.
2. Fawley MW, Fawley KP. A simple and rapid technique for the isolation of DNA from microalgae. J Phycol. 2004;40:223–5.
3. Nakada T, Nozaki H. Re-evaluation of three *Chlorogonium* (Volvocales, Chlorophyceae) species based on 18S ribosomal RNA gene phylogeny. Eur J Phycol. 2007;42:177–82.
4. Nozaki H, Ito M, Sano R, Uchida H, Watanabe MM, Kuroiwa T. Phylogenetic relationships within the colonial volvocales (Chlorophyta) inferred from *rbc*L gene sequence data. J Phycol. 1995;31:970–9.
5. Nozaki H, Ito M, Sano R, Uchida H, Watanabe MM, Takahashi H, Kuroiwa T. Phylogenetic analysis of *Yamagishiella* and *Platydorina* (Volvocaceae, Chlorophyta) based on *rbcL* gene sequences. J Phycol. 1997;33:272–8.
6. Felsenstein J. Confidence limits on phylogenies: an approach using the bootstrap. Evolution. 1985;39:783–91.
7. Tamura K, Stecher G, Peterson D, Filipski A, Kumar S. MEGA6: Molecular Evolutionary Genetics Analysis version 6.0. Mol Biol Evol. 2013;30:2725–9.
8. Nakazawa Y, Hiraki M, Kamiya R, Hirono M. SAS-6 is a cartwheel protein that establishes the 9-fold symmetry of the centriole. Curr Biol. 2007;17:2169–74.
